# Implementing a clinical assessment protocol for sensory and skeletal function in diabetic neuropathy patients at a university hospital in Brazil

**DOI:** 10.1590/S1516-31802005000500006

**Published:** 2005-09-01

**Authors:** Isabel de Camargo Neves Sacco, Silvia Maria Amado João, Denise Alignani, Daniela Kinoshita Ota, Cristina Dallemole Sartor, Leda Tomiko Silveira, Aline Arcanjo Gomes, Regeane Cronfli, Márcia Bernik

**Keywords:** Diabetes mellitus, Diabetic neuropathies, Disability evaluation, Physical Therapy Techniques, Touch, Diabetes mellitus, Neuropatias diabéticas, Avaliação da deficiência, Fisioterapia (técnicas), Tato

## Abstract

**CONTEXT AND OBJECTIVE:**

Physiotherapy can contribute towards recovering or preventing physical and sensory alterations in diabetic neuropathy patients. Our objective was to create and apply a protocol for functional assessment of diabetic neuropathy patients’ lower limbs, to guide future physiotherapy.

**DESIGN AND SETTING:**

Clinical study at the University Hospital and teaching/research center of Universidade de São Paulo.

**METHOdS:**

An intentional sample of diabetic neuropathy patients was utilized. The protocol was divided into: (1) preliminary investigation with identification of relevant clinical diabetes and neuropathy characteristics; (2) thermal, tactile and proprioceptive sensitivity tests on the feet; (3) evaluations of muscle function, range of motion, lower limb function, foot anthropometry.

**RESULTS:**

The patients’ mean age was 57 years, and they had had the diagnosis for 13 years on average. Distal numbness and tingling/prickling were present in 62% and 67%, respectively. There were tactile sensitivity alterations above the heel in 50%, with thermal sensitivity in 40% to 60%. The worst muscle function test responses were at the triceps surae and foot intrinsic muscles. Longitudinal plantar arches were lowered in 50%. Decreased thermal and tactile sensitivity of the heels was found. There was a general reduction in range of motion.

**CONCLUSIONS:**

The results provided detailed characterization of the patients. This protocol may be easily applied in healthcare services, since it requires little equipment, at low cost, and it is well understood by patients.

## RESUMO

**CONTEXTO E OBJETIVO:**

A fisioterapia pode contribuir na recuperação e prevenção nas alterações sensoriais e motoras em pacientes com neuropatia diabética (ND). Este protocolo foi criado e aplicado em diabéticos neuropatas clinicamente diagnosticados, com o objetivo de se avaliar funcionalmente membros inferiores (MMII), para futura indicação de tratamento fisioterapêutico.

**TIPO DE ESTUDO E LOCAL:**

Estudo clínico no Hospital Universitário e Centro de Docência e Pesquisa, Universidade de São Paulo.

**MÉTODOS:**

Amostragem intencional de diabéticos neuropatas foi utilizada. Protocolo: (1) investigação inicial: identificação de características clínicas relevantes da diabetes e da neuropatia; (2) testes de função muscular, amplitudes articulares, funcionalidade de MMII, antropometria dos pés; (3) avaliação das sensibilidades térmica, tátil e proprioceptiva dos pés.

**RESULTADOS:**

A média das idades dos pacientes foi de 57 anos, e eles apresentavam um tempo médio de diagnóstico clínico de diabetes de 13 anos. Adormecimento e agulhadas/formigamento estavam presentes em 62% e 67%, respectivamente. Alterações da sensibilidade tátil nos calcanhares foram percebidas em 50% dos pacientes, e da térmica, em 40% a 60%. As piores respostas dos testes de função muscular foram obtidas nos músculos tríceps sural e intrínsecos do pé. Os arcos longitudinais plantares estavam rebaixados em 50% dos pacientes. Houve redução geral nas amplitudes articulares.

**CONCLUSÕES:**

Os resultados possibilitam a caracterização dos pacientes avaliados. Este protocolo pode ser facilmente aplicado nos serviços de saúde, pois requer poucos equipamentos, é de baixo custo e é de fácil entendimento para os pacientes.

## INTRODUCTION

Diabetes mellitus can be considered to be a disease of epidemic proportions. According to the World Health Organization (WHO), Brazil has around six million diabetic individuals and is the country with the sixth largest number of people with diabetes.^1^ A multicenter study on diabetes mellitus prevalence in Brazil has estimated that 7.6% of the population aged 30-69 years has diabetes. Overall, there are 33.8 diabetics per 1000 inhabitants. In the 60 to 69-year-old group, 17.4% are diabetic.^2^

Peripheral neuropathy is the most insidious chronic complication of diabetes. Diabetic peripheral neuropathy refers to demonstrable disorders that are either clinically or subclinically evident and occur within the setting of diabetes mellitus without other causes for peripheral neuropathy. The neuropathic disorders involved include manifestations in the somatic and/or autonomic parts of the peripheral nervous system that can be noticed clinically and subclinically.^3^ Diabetic neuropathy can be classified as sensory, motor or autonomic, according to the degree and progression of its signs and symptoms. An international consensus meeting on the outpatient diagnosis and management of diabetic neuropathy has agreed on a simple definition consisting of the presence of symptoms and/or signs of peripheral nerve dysfunction in people with diabetes after the exclusion of other causes.^4^

Seventy-five percent of all diagnosed neuropathies are distal and symmetrical. Neuropathy usually leads patients to progressive loss of distal to proximal somatosensory sensitivity, especially during nighttime resting, and to the loss of distal proprioception and muscle and autonomic function.^5^ The prevalence of diabetic neuropathy five years after the diagnosing of diabetes is about 20%. Ten years after diagnosing, this prevalence rises to between 20 and 50%.^6,7^ Fifteen years after diagnosing, it has been found to be 40%.^5^ Furthermore, approximately 50% of diabetic patients over the age of 60 years show evidence of diabetic neuropathy.^8^

The common symptoms of diabetic sensory neuropathy turn progressively into motor neuropathy, typically with alterations in muscle trophism, especially in the intrinsic muscles of the feet and ankles. This can lead to deformation of the feet and reduction in the range of motion.^9^ The superficial nerves and the deep peroneal, surae and medial plantar nerves, in that sequence, are the first to be affected.^10,11^

Limitation of ankle mobility is prevalent among diabetic patients, in the subtalar or talocalcaneal joint and metatarsophalangeal joints, thereby increasing the risk of falls and injuries. Ankle dorsiflexion is significantly limited,^12–14^ because of a series of factors such as increased ankle rigidity, atrophy of the ankle extensor and tibialis anterior muscles, and alteration of the collagen structure in the fasciae and muscle tendons caused by the process of collagen glycosylation.^7^

There are no studies in the scientific literature that describe physiotherapeutic evaluation directed towards diabetic neuropathy patients. Considering the motor, sensory and functional damage caused by this disease, such evaluation becomes very important, in order to have better physiotherapeutic treatment for such patients. Physiotherapy can thus contribute in a singular way towards the recovery from or prevention of common physical and sensory alterations in these patients, through the use of an easily applied protocol for identifying and quantifying the skeletal and functional damage.

The difficulty in defining a single criterion for the diagnosis of neuropathy has been reviewed. Many researchers and professionals are unable to quantify symptoms, neurological deficits and peripheral nerve dysfunction tests because there are no specific protocols for this.^15^

In investigations regarding the early evaluation and diagnosis of diabetic neuropathy, researchers have been looking at losses of vibratory perception and reflexes in the distal lower limbs, and also abnormalities in nervous conduction in the sural nerve. It has been proposed that a complete clinical examination should include a combination of electrophysiological abnormality evaluations and somatosensory sensitivity tests.^10,16,17^ Other studies have demonstrated that a clearly defined personal interview, in association with clinical examination, can decisively diagnose diabetic neuropathy.^18^

Clinical examination, in association with the use of monofilaments to test the plantar surface tactile sensitivity, are the two most sensitive methods for identifying risks of ulceration among neuropathic patients.^9^ Such monofilament tests were used by Salsich and Mueller^19^ to confirm diabetic neuropathy. Monofilament tests are also recommended by the Consensus on the Diabetic Foot as an evaluation procedure for all diabetic patients.^20^

## OBJECTIVE

The aim of the present paper was to draw up and apply a protocol of physiotherapeutic evaluation that would allow identification of alterations to sensory and skeletal function among a group of clinically diagnosed diabetic neuropathy patients, in a cheap and practical manner that would be useful within our Brazilian reality, where the most expensive and modern equipment are not available. This physiotherapeutic evaluation would give specific directions for the creation of more efficient physiotherapy treatment for application to the chronic consequences of diabetic neuropathy.

## METHODS

The sampling method was intentional and led to the selection of 21 diabetic neuropathy patients of both sexes. These adult volunteers were outpatients at the interdisciplinary out-patient clinic for diabetic patients at the University Hospital of Universidade de São Paulo. Their diabetic neuropathy was confirmed by the hospital team from symptomatology and clinical investigation. The patients were selected by means of an interview that was based in a questionnaire drawn up previously from the literature. The inclusion criterion was that the subjects should be type 1 and 2 diabetic patients with symptoms related to diabetic neuropathy.^18^ The exclusion criteria were: age over 70; any neurological, muscle or rheumatic diseases outside of the diabetes etiology; or history of abusive alcohol intake. The subjects gave their agreement to participate in the study through signing a declaration of consent.

The ethical committee of the university hospital of Universidade de São Paulo approved the following experimental procedure: a) initial evaluation for characterization of the diabetic neuropathy;^17^ b) thermal, tactile and proprioceptive sensitivity evaluations on the soles of the feet; c) functional evaluation: muscle function tests, range of motion, anthropometric tests on the feet and functional testing of the lower limbs.

The tests that were used at each stage of the protocol are frequently used in scientific and clinical studies, and they have also already been validated.^7,17,20–25^ The tests were applied in an appropriate private room and, at that time, the patients described the status of their glycemic control.

The first stage of protocol consisted of the intentional selection of the 21 diabetic neuropathy patients by means of a screening interview based on a questionnaire. Diabetic neuropathy was characterized as present when at least two affirmative answers were given, in accordance with Feldmann et al.^18^

In the second stage of the protocol, patients’ tactile sensitivity was evaluated using a group of Semmes-Weinstein nylon monofilaments in five areas of the sole of the foot (hallux, heel, mid-foot, lateral forefoot and medial forefoot). Three monofilaments were used: 4.17, 5.07, and 6.10. Although the Diabetes Consensus^3^ establishes that sensitivity to the 5.07 monofilament is the criterion for diabetic neuropathy, it was decided to begin the test by using the 4.17 monofilament, which may indicate loss of both the protective sensitivity (meaning that the foot is thus more vulnerable to injuries) and the discriminatory sensitivity for hot and cold stimuli. Patients were considered to have normal tactile sensitivity if they were able to sense the 4.17 monofilament.

Proprioception assessment was carried out by means of the finger position perception test.^21^ The number of successes obtained by the patient after three attempts was recorded for analysis. Patients were considered to have normal proprioception function if they succeeded three times out of three attempts. The thermal sensitivity of the plantar surface was evaluated by means of contact with a hotand-cold 8-mm diameter body probe in five areas of the sole of the foot. Three touches were performed with the body probe on each plantar area, for the patient to identify the temperature at a single attempt. If the patient was able to correctly distinguish hot and cold stimuli, the patient's thermal sensitivity was considered normal.

At the functional evaluation stage, Kendall's muscle tests were used.^24^ This method assesses the function of specific muscles and requires selective muscle action. Muscle function testing was performed for the feet muscles and the ankle flexor and extensor muscles.

We used a universal goniometer^26^ to investigate joint range of motion, considering that this is the equipment most frequently used in physiotherapy evaluations. Ankle range of motion, knee flexion and extension and hip flexion were evaluated according to the criteria of Marques^22^ and Magee.^27^ Results from the evaluations of joint range of motion were compared with reference values in protocols already established.^22,27^

Also in this functional stage, a functional evaluation of the lower limbs was carried out by means of the tests described by Palmer and Epler.^25^ These tests are very practical and easily reproducible and consist of foot inversion and eversion tests and ankle and toe flexion and extension tests. Each test has a scale relating to the number of movement repetitions: ‘absent functionality’, ‘little functionality’, ‘reasonable functionality’ and ‘normal functionality’.

For the anthropometric test on the feet, patients were asked to stand still on a specially designed “foot measurer” consisting of a rubber mat with a sheet of paper on it, fully covered by ink. The footprint image thus registered allowed calculation of the longitudinal arch index and classification of the subjects’ type of arch: claw, flat or normal foot.^23^

All data from each patient were obtained by the same examiner using the evaluation protocol. Demographic and anthropometric data, clinical characteristics and joint range of motion were expressed in terms of their central trend and error and their means and standard deviations. The other data obtained were expressed as percentages. These statistical parameters allowed complete description of the sample studied.

## RESULTS

The mean age of the 21 diabetic patients (42.8% male, 57.2% female) was 56.9 ± 11.5 years old. Their mean body mass index (BMI) was 27.6 ± 4.5 kg/m^2^ and they had had a diagnosis of diabetes for 13.1 ± 8.1 years. Three patients had diabetic type 1 and 18 had type 2. Sixteen were Caucasian, four were Africans and one was Asian. Only 33.3% of them were physically active. Their mean glycemia level was 170.8 (71.2) mg/dl.

In order to characterize the patients’ diabetic neuropathy, a questionnaire^17^ consisting of fifteen questions was applied to each subject. The average score among the study sample was 6, thereby classifying them as diabetic neuropathy patients. In addition, the symptoms and signs most mentioned by the subjects were typical of diabetic neuropathy patients:^7^ distal numbness was reported by 62% of the patients, tingling/prickling by 67%, dry foot skin by 38%. In 52% of the cases, the symptoms reported occurred more frequently during nighttime resting. Among the sample group, only 24% of the patients reported having ever known about their diabetic neuropathy.

In the assessment of thermal sensitivity, 40% to 60% of the patients could only distinguish one of the types (hot or cold) in the heel. In accordance with the progressive loss of tactile sensitivity that is typical of diabetic neuropathy, only 50% of the patients did not respond to tactile stimulation on the heel with the 4.17 filament: these patients only sensed the stimulation by the 5.07 monofilament. However, the results from the proprioception test were normal for approximately 95% of the diabetic neuropathy patients assessed.

The results from muscle function testing showed that 40% to 50% of the patients reached grade four bilaterally in the following muscles: extensor digitorum longus, extensor digitorum brevis, interossei and triceps surae. In the same test, the flexor digitorum brevis and lumbricals of the foot showed grade four for 30-40% and 30-35% of the patients, respectively, thus demonstrating slight loss of muscle strength.

The results from the lower limb range of motion testing (Table 1) show greatly decreased foot inversion (0° to 18°/20°), when the normal would be from 0° to 40°.^24^ Dorsiflexion movement also became reduced, to the range of 0° to 14°. The subjects’ ankle plantar flexion also presented reduced width: 0° to 28°/30° (Table 1). Among these patients, 50% presented grade four in muscle function testing of the triceps surae, which is the main agent for ankle plantar flexion. The measurements of foot eversion, which present an average 0° to 11°/12° range of motion,^21^ found 8° for this movement performed by diabetic neuropathy patients.

**Table 1.** Mean and standard deviation of lower limb joint movement amplitudes, in degrees, in diabetic patients

Joint movement	Right	Left
Ankle plantar flexion (0 - 45°)	30.4 ± 10.0	28.6 ± 10.8
Ankle dorsiflexion (0 - 20°)	14.2 ± 5.3	14.9 ± 7.1
Ankle inversion (0 - 40°)	19.5 ± 6.5	18.0 ± 6.5
Ankle eversion (0 - 20°)	11.5 ± 4.1	12.4 ± 5.5
Knee flexion (0 - 140°)	127.7 ± 10.1	127.6 ± 9.7
Hip flexion (0 - 125°)	104.0 ± 15.3	104.1 ± 12.8

In the functional assessment,^25^ symmetry was noticed in the functional loss at the lower extremity, with full affection of dorsiflexion and eversion of the feet. Data for the testing of range of motion and degree of muscle function are shown in Table 2.

**Table 2.** Functional evaluation scores for the neuropathic diabetic patients

Side	Right (%)				Left (%)			
Functional score	1	2	3	4	1	2	3	4
Standing on one leg, with ankle dorsiflexion	40.0	0	15.0	45.0	52.4	0	14.3	33.3
Standing on one leg, with ankle plantar flexion	5.0	5.0	30.0	55.0	9.5	0	33.3	52.4
Standing on one leg, with ankle eversion	45.0	0	5.0	45.0	42.9	0	4.8	47.6
Standing on one leg, with ankle inversion	15.0	5.0	0	80.0	19.0	0	9.5	71.4
Sitting, with toe flexion	0	0	0	100	0	0	0	100
Sitting, with toe extension	0	0	10.0	90.0	0	4.8	9.5	85.7

Functional score: 1 = non-functional; 2 = slightly functional; 3 = reasonably functional; 4 = functional.

In the anthropometric evaluation, flattening of the longitudinal medial and lateral plantar arches was observed in 50% of patients’ right feet and in 33% of left feet. This alteration of foot structure represents a collapse of the plantar arches.

## DISCUSSION

The mean glycemia levels of the diabetic patients in the present study was greater than expected for diabetes control, since normal values are less than 126 mg/dl if based on the fasting plasma glucose test, or less than 140 mg/dl if based on the oral glucose tolerance test.^28^ One of the factors that predisposes towards diabetic neuropathy is high and fluctuating glycemia values. Poor metabolic control of glycemia is one of the most important factors linked to the development of diabetic neuropathy.^29^

The symptoms and signs most reported in the literature^7^ as typical of diabetic neuropathy patients are distal numbness, tingling/prick-ling and dry foot skin, and such patients have reported that these symptoms are more frequent during nighttime resting.

In the assessment of thermal sensitivity, patients could only distinguish one type of stimulus (hot or cold) in the heel. This area contains a larger amount of keratin and fat, and receives sensitivity innervation from the sural nerve. It is known that this nerve is one of the first to be damaged during the progression of diabetic neuropathy,^10,11^ and therefore the association of these two factors explains the decrease in thermal perception in the heel. Asymmetry between the right and left sides was noticed in characterizing this sensitivity, and this may indicate unilateral neuropathy. In fact, this corresponds to a classification of diabetic neuropathy found in the literature.^5^ The notable affection of the heel area seen in this type of sensory test using monofilaments represents loss of protective feeling, susceptibility to injuries and loss of hot/cold discriminatory power in this area.

The results from the proprioception test were approximately normal for the diabetic neuropathy patients assessed. In fact, reports in the literature often question the validity and effectiveness of clinical tests for evaluating proprioception in the foot.^21^ One hypothesis that would explain these results is based on the progressive pattern of diabetic peripheral neuropathy, which goes from distal to proximal limb damage, and from small caliber nonmyelinated nerve fibers to large caliber myelinated fibers. In this light, proprioception would be the last characteristic to be affected among somatosensory types, since this corresponds to the myelinated fibers. It may therefore be that these patients did not present this damage yet.

In the present study, alterations in the function of the triceps surae were observed, in accordance with findings in the literature.^30^ Such alterations may be due to physical inactivity (as reported by the patients), to the use of inadequate footwear, as observed in a similar population^31^ and to the diabetic neuropathy itself. All of these end up causing significant loss of muscle mass and, consequently, alterations in muscle function. When such losses affect the intrinsic muscles of the foot^5^ (interossei and ankle plantar flexor), which are responsible for maintaining foot structure, the latter can become deeply deformed, thus leading to alteration of the plantar arch.^30^

In range of motion testing of the lower limbs, according to the literature, an average inversion of 17° and dorsiflexion of 7° is expected among diabetic neuropathy patients,^21^ and the findings in the present study were similar to these. Also, significantly decreased ankle dorsiflexion has also been found.^14^ These movements may become reduced because of joint rigidity, which in turn is due to collagen structure alteration,^14^ or also because of loss of strength and trophism of the muscles involved in these joint movements. These factors have been reported to affect motion in diabetic neuropathy cases.^14^ Alternatively, the reduced range of motion can be attributed to the decrease in somatosensory sensitivity, and especially tactile and vibratory sensitivity. The subjects’ ankle plantar flexion also presented reduction. This decrease may also be due to joint rigidity and loss of muscle strength. The reduced range of foot eversion may be due to damage in the peroneal muscles, which are innervated by the superficial peroneal nerve, one of the first to be affected during the progression of diabetic neuropathy.^10,11^

Several factors explain the alterations in the functional assessment: (a) reduction of muscle strength, already mentioned, and also as reported;^13^ (b) decreased muscle endurance, as described;^13^ (c) decreased tactile sensitivity; (d) loss of muscle mass; and (e) reduced range of motion, as was observed in the present study. Decreased functionality in the lower extremity is an indication of possible disabilities, mobility loss and home nursing care needs.^32^

The alteration in foot structure presented in the present study represents a collapse of the plantar arches and has also been observed in the literature.^14,33^ Weakness of the intrinsic foot muscles that stabilize the tarsal bones was observed. This may be related to the flattening observed in the patients’ feet, since these muscles, along with the tibialis anterior, peroneal, extensor digitorum longus and hallux, keep the plantar arches normal.^34^

There are no studies in the scientific literature describing physiotherapeutic evaluation directed towards diabetic neuropathy patients. The patients were advised to take up preventive proactive physiotherapy intervention, in order to minimize the motor consequences previously described and to recover functional losses. Physiotherapy can contribute towards recovering skeletal function and improving the conditions of the musculoskeletal system in diabetic neuropathy subjects, considering that such patients are likely to develop muscle, skeletal and joint damage, as well as alterations in overall and segmental mobility, which are known sensory and motor consequences of diabetic neuropathy.

## CONCLUSIONS

The patients assessed by means of the protocol here described presented significant overall decrease in their range of motion, particularly in foot inversion and ankle bilateral dorsiflexion. Among their muscle functions, greater alterations were found bilaterally in the triceps surae muscle and the intrinsic foot muscles, probably due to the lowered longitudinal plantar arch. The alterations in muscle function and range of motion can explain the strongly affected bilateral functionality found in the lower limbs, especially the non-functionality in ankle dorsiflexion and eversion. The data obtained allowed good characterization of the diabetic neuropathy, which reinforces the applicability of this protocol for assessing neuropathy and the related skeletal damage.

The protocol here presented may be easily applied in healthcare services, since it requires little equipment, at low cost. It can be said that it was well understood by the patients, and it can be applied in one hour, which is enough time to get a sensory and musculoskeletal ‘picture’ of diabetic neuropathy patients’ conditions. Using this functional ‘picture’, we can devise specific and effective physiotherapy treatment for each patient. Our evaluation proposal presents a reproducible sequential method of sufficient extent to evaluate muskuloeskeletal and sensitivity functions in diabetic patients.
